# Investigation of the causal relationship between patient portal utilization and patient’s self-care self-efficacy and satisfaction in care among patients with cancer

**DOI:** 10.1186/s12911-024-02837-0

**Published:** 2025-01-08

**Authors:** Jaeyoung Park, Shilin Guo, Muxuan Liang, Xiang Zhong

**Affiliations:** 1 School of Global Health Management and Informatics, University of Central Florida, Orlando, FL USA; 2 Department of Industrial Engineering and Operations Research, Columbia University, New York City, NY USA; 3 Department of Biostatistics, University of Florida, Gainesville, FL USA; 4 Department of Industrial and Systems Engineering, University of Florida, 482 Weil Hall, PO BOX 116595, Gainesville, FL 32611-6595 USA

**Keywords:** Cancer care, Patient portal, Causal inference, Health self-efficacy

## Abstract

**Objective:**

The objective of this study was to examine the causal relationship between the usage of patient portals and patients’ self-care self-efficacy and satisfaction in care outcomes in the context of cancer care.

**Methods:**

The National Institute’s HINTS 5 Cycle 1–4 (2017–2020) data were used to perform a secondary data analysis. Patients who reported being ever diagnosed with cancer were included in the study population. Their portal usage frequency was considered as an intervention. Patient’s self-care self-efficacy and satisfaction in care were the primary outcomes considered and they were measured by survey respondents’ self-reported information. A set of conditional independence tests based on the causal diagram was developed to examine the causal relationship between patient portal usage and the targeted outcomes.

**Results:**

A total of 2579 were identified as patients with cancer or cancer survivors. We identified patient portals’ impact on strengthening patients’ ability to take care of their own health (*P* = .02, for the test rejecting which is necessary for the expected causal relationship, ie, the portal usage impacts the target outcome;* P* = .06, for the test rejecting which is necessary for the reverse causal relationship), and we identified heterogenous causal relationships between frequent patient portal usage and patients’ perceived quality of care (*P* = .04 and *P* = .001, for the tests rejecting both suggests heterogeneous causal relationships). We could not conclusively determine the causal relationship between patient portal usage and patients’ confidence in getting advice or information about health or cancer care related topics (*P* > .05 for both tests, suggesting inconclusive causal directions).

**Conclusions:**

The results advocate patient portals and promote the need to provide better support and education to patients. The proposed statistical method exploits the potential of national survey data for causal inference studies.

**Supplementary Information:**

The online version contains supplementary material available at 10.1186/s12911-024-02837-0.

## Background

Cancer is a major public health problem worldwide and is one of the six leading causes of death in the United States [1]. Many challenges exist for caring patients with cancer, including poor integration of survivorship care between the oncology and primary care settings, clinician workforce shortages and knowledge gaps about the needs of cancer survivors, and financial and other barriers to quality care, particularly among the medically underserved [2]. To address these challenges, ongoing efforts to identify best practices for the delivery of quality cancer rehabilitation and post treatment cancer care are needed. Effective patient-clinician communication has been identified as being associated with improved self-care [3], adherence to medications, pain control, and having a significant impact on patient satisfaction [4, 5], recall of information [6], and patient safety [7]. Patient-clinician communication is particularly important in cancer care, where research has shown that clinicians’ communication with patients empowers patients, impacts their psychosocial outcomes, enhances therapeutic alliances, and contributes to higher quality medical decisions [8, 9].

Patient portals, secure online websites linked to patients’ personal health records, are expected to enable such communication. The ability for patients to access personal health information and ask questions electronically about their treatment can facilitate improved communication, making them more engaged and activated about their health. Patient portals, thus, have attracted substantial attention, and summary of existing patient portal research can be found in systematic reviews [10–17]. Patients and healthcare providers alike showed interest in using portals for communication [13, 18–20], and patients used portal messaging to communicate needs and concerns [21]. This portal activity has been found to have the potential to improve communication [13–15] and patient satisfaction [16, 17, 22–24].

Patients with cancer rank the importance of having access to medical information higher than other patients because of the chronic nature of their care [25]. Subsequently, they use patient portals far more frequently than other patient populations [26] (pp2017-2018). The existing studies have demonstrated that patient portal usage was associated with improved care engagement and self-management capabilities across the cancer continuum [27], improved cancer-related outcomes [28], and high patient-centered communication among patients with cancer [29]. However, most studies in literature drawn association instead of causation, limiting their practical implications. On the other hand, clinical trials to investigate the effects of patient portals among patients with cancer might not be scalable due to low patient participation [27].

Casual discovery using observational data has gained growing attention in the public health field where large-scale randomized controlled trials are infeasible. A general survey on causal discovery can be found in Zanga et al. [30] and a survey of Bayesian Network structure learning can be found in Kitson et al. [31]. Notably, Pearl’s causal diagram identifies causal directions using d-separation, which involves conditional independence tests [32]. These tests check if potential backdoor paths between the outcome and the intervention are blocked by conditioning on certain confounders. This causal diagram can accommodate various types of variables, including an intervention, an outcome, confounders, and an instrumental variable (IV) [33].

Building upon extant literature, this study aimed to investigate the causality between patient portal usage and patients’ self-care self-efficacy and satisfaction in care among patients with cancer. As defined in the literature [34], self-care self-efficacy is an individual's belief in their capacity to perform self-care behaviors. The concept of self-care is multidimensional and includes self-responsibility and health information-seeking behavior. Health information-seeking behavior (HISB), also known as health information seeking, refers to a series of interactions that reduces uncertainty regarding health status and constructs a social and personal sense of health. The existing literature has shown that high self-efficacy in HISB is associated with improved cancer outcomes [35]. In our previous study using the Health Information National Trends Survey (HINTS) data that regularly collects nationally representative data about the American public’s knowledge of, attitudes toward, and use of cancer- and health-related information [36], we have analyzed the causal effects of portals among general survey participants and concluded that patients who actively utilize their patient portal experience improved self-reported outcomes, such as patients’ confidence in obtaining health information, compared to those using it less actively [37]. Due to its unique patient portal utilization patterns, this study focused on the cancer population in HINTS data. Among patients with cancer, the goal was to investigate whether portal usage can enhance patients’ self-efficacious health information–seeking behaviors, their ability to exercise self-care, and perception of care quality, based on the survey responses.

## Methods

### Study population

Data for this cross-sectional study was obtained from the National Institute’s HINTS 5 Cycle1 (2017), Cycle 2 (2018), Cycle 3 (2019), and Cycle 4 (2020). According to the HINTS report [38], the number of respondents and response rate for each year were 3285 (32.4%), 3504 (32.9%), 5438 (30.3%), and 3865 (36.7%) for HINTS 5 Cycles 1–4, respectively. The population of interest was patients with cancer and cancer survivors, made up of those who answered “Yes” to the question “Have you ever been diagnosed as having cancer?” Over the four years of data, this segment had a total of 2579 participants in the HINTS questionnaire, accounting for 16.1% of the pooled data population (*N* = 16092).

### Relevant measures

Each cycle of HINTS 5 data includes questions about patient portal usage and Internet access in the last 12 months since the survey was distributed. The survey also assesses patient factors including health care access (eg, insurance coverage), their digital literacy (eg, use of Internet to view health information), demographic details (eg, income), and health characteristics (eg, comorbidities). The characteristics of the patient factors that are considered as confounders in this study are age, gender, race, ethnicity, education, marital status, income, and insurance type. In medical research, these factors are commonly used as confounders that are both associated with technology adoption and health outcomes [39–41]. The distributions of these factors across different portal usage frequencies are summarized in Table S1 in Supplementary Material 1.

Patient’s self-care self-efficacy and satisfaction in care were the primary outcomes considered in this study, and they were measured by respondents’ answers to the following questionnaire items in HINTS. These items are commonly used as outcome measures in the literature [42]:

1. “Overall, how confident are you about your ability to take good care of your health?” (OwnAbilityTakeCareHealth [OATCH], Y1)
2. “Overall, how would you rate the quality of health care you received in the past 12 months?” (QualityOfCare [QC], Y2)
3. “Overall, how confident are you that you could get advice or information about health or medical topics if you needed it?” (ConfidenceGettingHealthInfo [CGHI], Y3)
4. “Overall, how confident are you that you could get advice or information about cancer if you needed it?” (ConfidenceGettingCancerHealthInfo [CGCHI], Y4)

Responses were recorded with a 5-point Likert-type scale, and Table S3 in Supplementary Material 1 displays the possible responses for each question.

The patient's portal usage was considered as the intervention and was assessed with the questionnaire item: “How many times did you access your online medical record in the last 12 months?” with options being ‘0 = 0’, ‘1 = 1 to 2 times’, ‘2 = 3 to 5 times’, ‘3 = 6 to 9 times’, ‘4 = 10 or more times’. Based on usage frequencies, we defined five different levels of intervention, instead of treating intervention as a binary variable. This allows us to understand the intensity of portal usage and its impact on the outcomes.

We used Internet savviness as the IV assessed based on the questionnaire items: “How often do you access the Internet through each of the following?” with four specific venues, including “computer at home”, “computer at work”, “computer in public place (library, community center other)”, or “on a mobile device (cell phone/ smartphone/ tablet).” If a participant responded “Daily” to any of the four items, we regarded this participant as Internet savvy, which indicated a better access to the patient portal. The qualification of this instrument variable is justified in the Results section.

### Statistical analysis

To identify causal relationship using the national survey data, we have proposed a testing framework based on Pearl’s causal diagram [37]. In the proposed framework, we adopted an IV to learn the structure of the graph to inform the direction of the causal relationship between two variables of interest, even if there is a lack of temporal information [43, 44]. This IV also adjusts for both observed and unobserved confounding effects, obviating the need to exclusively identify all confounders. Thus, the proposed framework can accommodate measured confounders (e.g., patients’ demographic and socioeconomic status) and unmeasured confounders. This approach was developed under the assumption that all individuals in the population present the same causal relationship. However, due to the heterogeneity of the population, it is possible that the causal relationship is also heterogeneous across different subgroups. In this work, we further extend this approach to test the causal relationship among several pre-specified subgroups. This approach assumes that different subgroups may have different causal relationships.

**Fig. 1:**
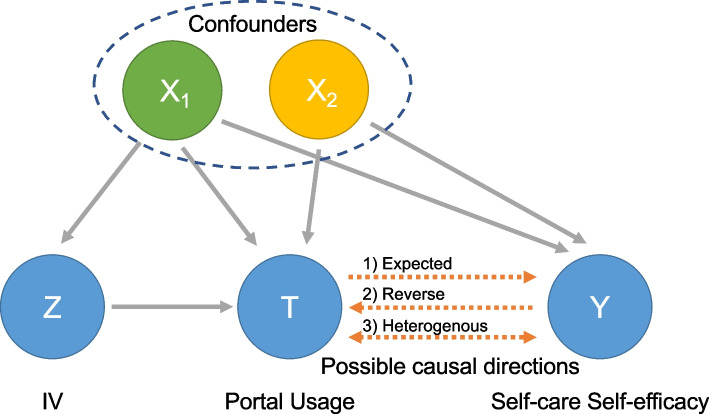
The potential causal relationship between portal usage and self-care self-efficacy. Abbreviations: IV, Instrumental Variable

In our analysis, we introduced Internet savviness as an IV. We examined the associations between the Internet savviness and the intervention (patient portal usage behavior) to justify the qualification of using Internet savviness as an IV. Next, we examined the associations between the intervention and each patient outcome. Although the association of patient portal usage and some of the selected outcomes have been discovered, the directional causal relationship is unsettled [45]. It is unclear whether patient portal usage causes an improvement in the outcome, or vice versa. Both directions might exist due to the heterogeneity within a population. For certain individuals, using a patient portal usage could improve the outcome, whereas, for others, patients’ self-care self-efficacy might lead to increased portal usage. The graph to represent the potential causal relationship is shown in Fig. 1.

To address the confounding issue and determine the direction of the causal relationship, we tested two hypotheses for the whole population based on the graph model: 1) whether $T$ causes $Y$, also known as the expected causal direction; 2) whether $T$ is caused by $Y$, ie, the reverse causal direction. The condition of no confounding between $Z$ and $Y$ cannot be easily justified in observational studies, and the judgment highly depends on the domain knowledge. A more realistic assumption is to allow for some known part of confounders (denoted as ${X}_{1}$) to contribute to the confoundedness between Z, $T$, and $Y$. Therefore, in our causal analysis, we further generalize the criteria of IVs to allow for known (or observable) confounding among $Z$, $T$, and $Y$ and unknown (or unobservable) confounding between $T$ and $Y$. Consequently, we split confounders $X$ into two disjoint groups, ${X}_{1}$ and ${X}_{2}$, as illustrated in Fig. 1. Variables in ${X}_{1}$ are the known common confounders between $Z$, $T$, and $Y$. Variables in ${X}_{2}$ are the confounders between $T$ and $Y$, and ${X}_{2}$ is not required to be fully measurable. If $Z$ is independent with $X$, then ${X}_{1}$ is an empty set, and the causal diagram degenerates to the classical IV framework.

As described in [37], the causal direction could be determined by using the following two conditional independence tests: 1) one between $Z$ and $Y$ conditioning on ${X}_{1}$, ie, $\text{Z}\perp \text{Y }|{\text{X}}_{1}$; 2) the other between $Z$ and $Y$ conditioning on ${X}_{1}$ and $T$, ie, $\text{Z}\perp \text{Y }|({\text{X}}_{1}, \text{T})$. If and only if $T$ causes $Y$ exclusively for the whole population, then, $Z$ and $Y$ are conditionally independent given ${X}_{1}$ and T, but not given ${X}_{1}$. If and only if the opposite direction is true for the whole population, then, $Z$ and $Y$ are conditionally independent given ${X}_{1}$, but not given ${X}_{1}$ and $T$. For the test efficiency, the conditions were modified as follows: $\text{Z}\perp \text{Y }|{\overline{\uppi } }_{{\text{X}}_{1}}$ (denoted as *Test B*) and $\text{Z}\perp \text{Y }|{\overline{\uppi } }_{{\text{X}}_{1},\text{T}}$ (denoted as *Test A*), where ${\overline{\uppi } }_{{\text{X}}_{1}}$ and ${\overline{\uppi } }_{{\text{X}}_{1},\text{T}}$ are the discretized version of the propensity scores ${\pi }_{{X}_{1}}=\text{P}\left(\text{Z}=1|{X}_{1}\right),$ and ${\pi }_{{X}_{1},T}=\text{P}(\text{Z}=1|{X}_{1},\text{T})$. We explored the robustness against different discretization using numerical experiments and discretized the propensity score based on five strata (see more details in Supplementary Material 1).

Further, due to the heterogeneity of the population, it is possible that the causal relationship is also heterogeneous across different subgroups. As an example, it is not unreasonable to speculate that using patient portal frequently will lead old people (or people with poor health literacy) to improve their confidence to get health information, ie, the causal direction from $T$ to $Y$. Meanwhile, in the younger population (or people with better health literacy), because they are quite confident about using Internet to search health information, they are customized to use patient portals and might prefer to use it more (the causal direction from $Y$ to $T$). Such reverse causation has been reported in other medical fields [46].

To explore the possibility that the general population is a compound of subgroups that exhibit heterogenous causal relationships, not just in the level of impact (ie, the magnitude of the causal effect), but also in the direction of the impact (ie, the direction of the causal relationship), we proposed further test procedures. It is possible that both tests (ie, Test A and Test B) can be rejected or not rejected at the same time when applying the tests to the whole population. For the non-rejection case, it is likely because the sample size is not large enough to detect the difference in distributions. Meanwhile, the rejection case suggests that, the causal relationship is not universal in the target population. Therefore, when both Test A and Test B are rejected, an investigation into patient subgroups is performed. Within each subgroup, Tests A and B are performed to investigate the causal relationship therein. The subgroups can be determined based on prior knowledge. In this study, we chose education level [47, 48] and gender [15, 49], both have been shown to be associated with disparity in patient outcomes and patient portal usage behaviors.

There are two approaches to implementing the conditional independence tests: Cochran-Mantel–Haenszel (CMH) Test [50], which includes the Chi-square test, and Conditional Mutual Information [51]. Mutual information is an information-theoretic distance measure, which can be used to test for conditional independence. It is proportional to the log-likelihood ratio and is related to the deviance of the tested models [52]. When all the involving variables (i.e., the intervention, the outcome, and the propensity score) are discrete, the CMH test and Conditional Mutual Information are linked in the context of the conditional independence test [53]. We adopted the Conditional Mutual Information approach which is well-implemented in the R Bnlearn package [54].

Missing values were handled in several ways depending on the variable type: samples missing the outcomes of interest were discarded for the study of each outcome; missing covariates (confounders) were imputed using the Multivariate Imputation by Chained Equations (MICE). To reduce the bias from missing imputation, we imputed missing covariates 100 times with different random seeds, conducted the conditional independence tests using each imputed dataset, and reported the pooled *P* value, instead of a single *P* value [55–57].

### Software

In this study, all statistical analyses were conducted in R programming. We used the mice package (version 3.14.0) for the missing imputation and the Bnlearn package (version 4.7) for Conditional Mutual Information to perform the conditional independence tests [54].

## Results

We included respondents who reported a personal history of cancer and at least one of the instrumental variable related questions, and the sample size was 2579 (16.1% of the pooled data, *N* = 16,092). More female (female: *n* = 1487, 57.7%; male: *n* = 1065, 41.3%) and elderly (65 + : *n* = 1597, 61.9%) patients responded the survey, and White dominated the study population (White: *n* = 1999, 77.5%; Black or African American: *n* = 298, 11.6%). Table S1 in Supplementary Material 1exhibits respondents’ characteristics in the column of Total.

Patient portal utilization patterns differed in demographic and socioeconomic status (see Table S1 in Supplementary Material 1). Patients aged 75 years or older used patient portals the least, while those in the middle-aged group (35–49) used portals the most. Compared to White patients, Black or African American patients used portals remarkably less. Approximately three-quarters (73.3%) of patients with the lowest income group ($0—$19,999) never accessed patient portals for the last 12 months, significantly lower than those in the other income groups. For insurance, Medicare and Medicaid beneficiaries utilized portals less than patients with health insurance supported by employers. These variations in portal behaviors justified the use of demographic and socioeconomic factors as observed confounders.

### Association analysis

We first examined the relationship of the intervention (patient portal usage behavior) and the IV (Internet savviness). Of the 2579 participants, we identified 1596 (61.9%) as Internet savvy. Among the 1596 Internet savvy patients, 709 (44.4%) were non-portal users, 284 (17.8%) patients used the portal 1–2 times in a year, 312 (19.5%) 3–5 times, 124 (7.8%) 6–10 times, and 135 (8.5%) used the portal more than 10 times in a year, 32 (2.0%) did not respond to the question regarding portal usage. Among the remaining 983 (38.1%) patients who were not identified as Internet savvy, the corresponding numbers were: 769 (78.2%), 58 (5.9%), 52 (5.3%), 20 (2.0%), 20 (2.0%), and 64 (6.5%). The Chi-square test yielded a small *P* value (< 0.001) that suggests a strong association between the intervention and the IV (see Table S2 in Supplementary Material 1).

We further examined the relationship between the intervention and the outcomes of patients’ ability to take care of their own health, to obtain health information/cancer care information, and perception of quality of care. Chi-square tests were performed, and the results suggested a strong associative relationship between the intervention and all the outcomes (see Table S3 in Supplementary Material 1).

### Causal relationship between patient portal usage and targeted outcomes

We first performed tests on the general cancer population. Test A tests whether higher self-care self-efficacy or satisfaction of quality of care is a cause for increased portal usage. Test B examines whether the increase in portal usage is a cause for higher self-care self-efficacy or satisfaction of care quality. A *P* value < 0.05 was used as the statistical significance level for these tests. The effect size of a Chi-squared test was obtained by Cramer’s V and evaluated by Cohen’s guideline [58]. Table 1 presents the test results using the Conditional Mutual Information method [54].

**Table 1:** Results of conditional independence tests for the unstratified cancer population

	Test A^a^		Test B^b^	
Outcomes^c^	*Pooled P* value	Effect size	*Pooled P* value	Effect size
OATCH –﻿ OwnAbilityTakeCareHealth	.06	.10	.02	.11
QC –﻿ QualityOfCare	.046	.10	.004	.13
CGHI –﻿ ConfidenceGettingHealthInfo	.10	.12	.07	.13
CGCHI – ﻿ConfidenceGettingCancerHealthInfo	.34	.10	.11	.15

^a^Rejecting Test A is necessary for the reverse causal relationship

^b^Rejecting Test B is necessary for the expected causal relationship

^c^Failing to reject Test A but rejecting Test B indicates existence of the expected causal relationship between the treatment and the outcome; the opposite indicates the reverse causal relationship. Rejecting both tests indicates existence of heterogenous causal relationships

For OATCH, we could not reject the null hypothesis for Test A (*P* = 0.06) but could reject the null hypothesis for Test B (*P* = 0.02), meaning that portal usage did have a causal relationship on the patients’ confidence in exercising self-care. According to Cohen’s guideline [58], the effect size values for both tests suggested low to moderate practical significance. It should be noted that sample sizes (see Table S3 in Supplementary Material 1) are different for each outcome due to different response rates. For CGCHI and CGHI, we could not reject the null hypothesis for either Test A or Test B, which means that we could not determine the causal relationship between the patients’ portal usage and their confidence in obtaining health or cancer related information. For QC, however, we could reject the hypothesis for both Test A and Test B at 5% level of significance, which motivated a sub-group analysis for that outcome and the results are presented in Table 2.

**Table 2:** Subgroups analysis for potential causal relationships

Outcome^c^ – Quality of Care	Test A^a^		Test B ^b^	
Subgroups	*Pooled P* value	Effect size	*Pooled P* value	Effect size
Gender				
Female	.17	.12	.02	.15
Male	.28	.13	.16	.14
Education				
equivalent or lower than college graduates	.01	.15	.06	.14
graduate or postgraduate	.76	.10	.12	.15

^a^Rejecting Test A is necessary for the reverse causal relationship

^b^Rejecting Test B is necessary for the expected causal relationship

^c^Failing to reject Test A but rejecting Test B indicates existence of the expected causal relationship between the treatment and the outcome; the opposite indicates the reverse causal relationship. Rejecting both tests indicates existence of heterogenous causal relationships

As an illustrative example, we considered different gender and education groups. For the subgroup whose highest level of education is equivalent or lower than college graduates (high school, in college or vocational training etc.), we could reject the null hypothesis for Test A (*P* = 0.01) but could not reject the null hypothesis for Test B (*P* = 0.06), suggesting that the perceived quality of care received has causal relationship with their usage of the patient portal. Meanwhile, for the higher education group (graduate or postgraduate), we did not have sufficient samples to draw conclusions. In contrast, when we separated patients into different sex groups, we could not reject the null hypothesis for Test A (*P* = 0.17) but reject the null hypothesis of test B (*P* = 0.02), suggesting that the portal usage has causal relationship with patients’ perception of the quality of health care received for the female group. All the effect sizes indicated low to moderate practical significance. Together these results support the hypothesis that mixed causal relationships could exist in the general population. Note that these results do not suggest that gender or education are the only factors that contribute to the heterogeneous causal relationship.

## Discussion

In this study, we identified causal relationship between patient portal usage and patients’ perception regarding their ability to take care of their own health and the quality of care they received among patients with cancer based on their HINTS survey responses. Extant studies for patient portal or patients with cancer using HINTS data have been focused on the disparity in patient portal adoption [59], the trend in patient portal usage [40], the relationship between online health record access and patient-provider communication [60], and factors influencing health information seeking behaviors [61]. However, there are very few studies that investigate the causal relationship between patient portal usage and patient’s self-care self-efficacy and satisfaction of their care. Choudhury et al. [62] analyzed the relationship between patients’ understanding of online medical records and their perception of care quality, and a positive association was identified. In this study, we further confirmed the *causal relationship* of patient portal usage using the HINTS dataset, which provides stronger evidence of the benefit of portal usage. Additionally, the proposed testing procedure allows for the detection of heterogenous causal relationships in observational data when the temporal information is ambiguous. Major concerns of causal inference using cross-sectional data include the existence of heterogenous causal relationships and the lack of temporal information, as discussed in the literature [63]. Our method enables the exploration of survey data like HINTS to establish causal relationships and supports a deeper understanding of the possible complex causal relationships in a diverse study population.

### Effect of portal usage to improve patient outcomes

The observations from this research bolster the evidence of patient portals’ impact on strengthening patients’ ability to take care of their own health. This is possibly attributed to patient portals’ ability to empower patients. For example, oncologists have noted that patients using the portal were more participatory than patients who did not use the portal [64].

Furthermore, when patients are able to effectively communicate and be active participants in their care, they are significantly more likely to feel their treatment plans reflect their values [65]. We observed that the use of patient portals can improve female patients’ perception of the quality of health care received among patients with cancer, while the investigation is inconclusive for the male counterpart. Interestingly, when we look at the population based on patients’ education level, the perceived quality of care received has causal relationship with their usage of the patient portal. This might suggest that patients receiving high quality of care are positive about their experience with their care providers and thus would like to use the patient portal more often. However, this was only observed in the population with an education level of college or below and it is inconclusive for the higher education group. Note that the female group and the lower education level group are not mutually exclusive. This warrants a future investigation into unique and more refined patient subgroups.

These findings will support the intervention effort to target patients who can benefit the most from using patient portals. For a certain segment of patients with cancer, encouragement of patient portal usage might not be sufficient to increase their perceived quality of care. Understanding heterogeneous treatment effects might help facilitate the development of effective intervention strategies. In our investigation, we noted comparable portal usage among male and female patients but discerned disparities in the enhancement of their self-care self-efficacy. This might imply that the current intervention strategy may exhibit a lesser impact on male patients. The divergence in portal behaviors between the two demographic groups may contribute to the observed variation in the outcome. Therefore, a more in-depth exploration utilizing detailed usage information of patient portal functions is warranted. Strategically tailored promotion and education of patient portal usage will be instrumental to its meaningful use and the improvement of all patients’ outcomes.

### Limitations

It has been reported that patient portals offer the opportunity to deepen relationships with patients with cancer by increasing transparency of health information and supporting communication [66]. In this study, we examined cancer participants’ responses to the question “how confident are you that you could get advice or information about cancer or general health and medical topics.” However, we were not able to draw a conclusion like we did for general survey participants regarding patient portals’ impact on this matter [37]. We expect more data to strengthen the statistic power of the analysis as patient portal interventions designed to stimulate communication for cancer care have been shown to be effective in encouraging patients to ask more questions, be more assertive, and express pain-related concerns [67].

Additionally, it has been reported that patients with cancer use patient portals far more frequently than other patient populations [26]. It would be of interest to understand if the additional use is attributed to managing cancer care, and whether it was the oncology-specific portal usage that directly impacts patient outcomes like self-care self-efficacy. Unfortunately, the distinction between general portal use and oncology-specific portal use is not available in the HINTS data. Such granular data might be accessible through individual health institutions [68], and a study using such data has the potential to precisely identify causal effects of oncology-specific portals on self-efficacy in cancer care.

For the proposed method, our current test framework is able to examine whether there exist heterogeneous causal relationships in the study population. However, it does not support identifying the exact subgroups that behave differently from others. Our current subgroup analysis is only based on one patient factor and is just for a proof-of-concept to show the different causal directions. It is worth noting that the proposed method cannot identify the factors that contribute to the heterogeneity. Detecting subgroups will be a challenge undertaking because multiple factors might contribute to these distinct behaviors and these factors might not be known upfront or accessible. Future efforts are needed to provide approaches that can effectively identify the distinct subgroups. Additionally, survey data typically suffer missingness and this is also the case in HINTS. In our study, we employed MICE as the imputation approach and confirmed its robustness through numerical experiments. There is a vast of studies on the impact of missing data on causal discovery [30, 57]. A future direction is to explore data imputation methods to ensure the proposed framework is robust against missing data in general applications. Finally, if the propensity score model is not properly specified, it can lead to biased estimates of the causal relationship. We presented an investigation into different propensity score models in the Supplemental Material (Table S5) and further validation approach is warranted due to lack of ground truth.

In terms of the findings, although we observed the causal relationship between the patient portal usage and several patient self-reported outcomes, we were not able to identify exactly what types of portal function (e.g., billing, messaging, prescription refill, viewing after visit summary, etc.) that improve patients’ health self-efficacy. This knowledge might help explain the heterogeneous causal relationships observed. For instance, it might be the use of messaging function alone that causes improved quality of care perceived and patients whose primary use of patient portals is not messaging might not show a significant improved care experience. A study investigating the specific patient portal functions might be able to provide further insights into the complex causal relationships.

## Conclusion

Our proposed statistical method exploits the potential of using national survey data such as the HINTS program to examine causal relationships to obtain new insights given the lack of randomized experimental data. The causal relationship can be heterogeneous based on different patient characteristics and the testing framework enables the identification of the disparity in causal relationships. Ultimately, this study can facilitate the development of a multi-level intervention that identifies patients who will benefit the most from using patient portals, educates clinicians about promoting the portal, and monitors portal activities.

## Supplementary Information

Supplementary Material 1.

## Data Availability

The datasets generated during and/or analyzed during this study are available in the HINTS repository (https://hints.cancer.gov/).

## Abbreviations

CGCHI: ConfidenceGettingCancerHealthInfo
CGHI: ConfidenceGettingHealthInfo
CMH: Cochran-Mantel-Haenszel
DAG: Directed Acyclic Graph
HINTS: Health Information National Trends Survey
HISB: Health Information-Seeking Behavior
HITECH: Health Information Technology for Economic and Clinical Health
IV: Instrumental Variable
MICE: Multivariate Imputation by Chained Equations
OATCH: OwnAbilityTakeCareHealth
QC: QualityOfCare
