# Supplementary material for: Investigation of the causal relationship between patient portal utilization and patient’s self-care self-efficacy and satisfaction in care among patients with cancer
Source: BMC Med Inform Decis Mak. 2025 Jan 8;25:12. doi: 10.1186/s12911-024-02837-0 (PMC11716468; doi:10.1186/s12911-024-02837-0)
Supplement: Supplementary file 1 — Supplementary Material 1. [file 12911_2024_2837_MOESM1_ESM.docx]

Table S1. Characteristics of cancer survey respondents stratified by portal usage levels. *P* values are the results of Chi-square tests for each characteristic excluding no responses.

| Characteristics of patients | Total | Portal usage level (times) | | | | | | *P* value |
| --- | --- | --- | --- | --- | --- | --- | --- | --- |
|  |  | Level 1 (0) | Level 2 (1 – 2) | Level 3 (3 – 5) | Level 4 (6 – 9) | Level 5 (10+) | No response |  |
|  |  | 1478 (57.3) | 342 (13.3) | 364 (14.1) | 144 (5.6) | 155 (6.0) | 96 (3.7) |  |
| **Age, n(%)** | | | | | | | | **< 0.001** |
| 18-34 | 36 (1.4) | 20 (55.6) | 5 (13.9) | 4 (11.1) | 3 (8.3) | 3 (8.3) | 1 (2.8) |  |
| 35-49 | 164 (6.4) | 73 (44.5) | 30 (18.3) | 33 (20.1) | 8 (4.9) | 17 (10.4) | 3 (1.8) |  |
| 50-64 | 727 (28.2) | 358 (49.2) | 121 (16.6) | 121 (16.6) | 47 (6.5) | 62 (8.5) | 18 (2.5) |  |
| 65-74 | 832 (32.3) | 461 (55.4) | 127 (15.3) | 128 (15.4) | 51 (6.1) | 47 (5.6) | 18 (2.2) |  |
| 75+ | 765 (29.7) | 527 (68.9) | 54 (7.1) | 73 (9.5) | 32 (4.2) | 26 (3.4) | 53 (6.9) |  |
| No response | 55 (2.1) | 39 (70.9) | 5 (9.1) | 5 (9.1) | 3 (5.5) | 0 (0.0) | 3 (5.5) |  |
| **Gender, n(%)** | | | | | | | | 0.756 |
| Male | 1065 (41.3) | 625 (58.7) | 134 (12.6) | 151 (14.2) | 61 (5.7) | 61 (5.7) | 33 (3.1) |  |
| Female | 1487 (57.7) | 835 (56.2) | 206 (13.9) | 212 (14.3) | 80 (5.4) | 94 (6.3) | 60 (4.0) |  |
| No response | 27 (1.0) | 18 (66.7) | 2 (7.4) | 1 (3.7) | 3 (11.1) | 0 (0.0) | 3 (11.1) |  |
| **Race, n(%)** | | | | | | | | **<0.001** |
| White | 1999 (77.5) | 1096 (54.8) | 288 (14.4) | 302 (15.1) | 118 (5.9) | 136 (6.8) | 59 (3.0) |  |
| Black or African America | 298 (11.6) | 205 (68.8) | 31 (10.4) | 22 (7.4) | 9 (3.0) | 11 (3.7) | 20 (6.7) |  |
| Asian | 50 (1.9) | 25 (50.0) | 4 (8.0) | 13 (26.0) | 2 (4.0) | 2 (4.0) | 4 (8.0) |  |
| Other | 100 (3.9) | 56 (56.0) | 15 (15.0) | 18 (18.0) | 5 (5.0) | 2 (2.0) | 4 (4.0) |  |
| No response | 132 (5.1) | 96 (72.7) | 4 (3.0) | 9 (6.8) | 10 (7.6) | 4 (3.0) | 9 (6.8) |  |
| **Ethnicity, n(%)** | | | | | | | | **0.003** |
| Hispanic | 183 (7.1) | 124 (67.8) | 18 (9.8) | 17 (9.3) | 11 (6.0) | 6 (3.3) | 7 (3.8) |  |
| Non-Hispanic | 2110 (81.8) | 1146 (54.3) | 312 (14.8) | 328 (15.5) | 120 (5.7) | 144 (6.8) | 60 (2.8) |  |
| No response | 286 (11.1) | 208 (72.7) | 12 (4.2) | 19 (6.6) | 13 (4.5) | 5 (1.7) | 29 (10.1) |  |
| **Education, n(%)** | | | | | | |  | **<0.001** |
| High school or less | 687 (26.6) | 505 (73.5) | 47 (6.8) | 55 (8.0) | 22 (3.2) | 26 (3.8) | 32 (4.7) |  |
| Some college | 780 (30.2) | 445 (57.1) | 120 (15.4) | 104 (13.3) | 50 (6.4) | 38 (4.9) | 23 (2.9) |  |
| College graduate | 567 (22.0) | 282 (49.7) | 87 (15.3) | 103 (18.2) | 34 (6.0) | 40 (7.1) | 21 (3.7) |  |
| Post graduate | 491 (19.0) | 210 (42.8) | 85 (17.3) | 99 (20.2) | 35 (7.1) | 49 (10.0) | 13 (2.6) |  |
| No response | 54 (2.1) | 36 (66.7) | 3 (5.6) | 3 (5.6) | 3 (5.6) | 2 (3.7) | 7 (13.0) |  |
| **Marital Status, n(%)** | | | | | | | | **<0.001** |
| Married | 1310 (50.8) | 678 (51.8) | 200 (15.3) | 223 (17.0) | 80 (6.1) | 92 (7.0) | 37 (2.8) |  |
| Divorced & Separated | 494 (19.2) | 291 (58.9) | 66 (13.4) | 67 (13.6) | 30 (6.1) | 28 (5.7) | 12 (2.4) |  |
| Widowed | 472 (18.3) | 326 (69.1) | 45 (9.5) | 42 (8.9) | 17 (3.6) | 12 (2.5) | 30 (6.4) |  |
| Single | 249 (9.7) | 148 (59.4) | 28 (11.2) | 27 (10.8) | 14 (5.6) | 21 (8.4) | 11 (4.4) |  |
| No response | 54 (2.1) | 35 (64.8) | 3 (5.6) | 5 (9.3) | 3 (5.6) | 2 (3.7) | 6 (11.1) |  |
| **Income, n(%)** | | | | | | | | **<0.001** |
| $0-$19,999 | 475 (18.4) | 348 (73.3) | 35 (7.4) | 31 (6.5) | 19 (4.0) | 20 (4.2) | 22 (4.6) |  |
| $20,000-$49,999 | 564 (21.9) | 244 (43.3) | 103 (18.3) | 115 (20.4) | 45 (8.0) | 46 (8.2) | 11 (2.0) |  |
| $50,000-$100,000 | 763 (29.6) | 486 (63.7) | 79 (10.4) | 92 (12.1) | 25 (3.3) | 42 (5.5) | 39 (5.1) |  |
| $100,000+ | 738 (28.6) | 369 (50.0) | 123 (16.7) | 126 (17.1) | 54 (7.3) | 47 (6.4) | 19 (2.6) |  |
| No response | 39 (1.5) | 31 (79.5) | 2 (5.1) | 0 (0.0) | 1 (2.6) | 0 (0.0) | 5 (12.8) |  |
| **Health Insurance – Employer, n(%)** | | | | | | |  | **<0.001** |
| Y | 1113 (43.2) | 553 (49.7) | 175 (15.7) | 191 (17.2) | 79 (7.1) | 86 (7.7) | 29 (2.6) |  |
| N | 1379 (53.5) | 876 (63.5) | 163 (11.8) | 163 (11.8) | 62 (4.5) | 69 (5.0) | 46 (3.3) |  |
| No response | 87 (3.4) | 49 (56.3) | 4 (4.6) | 10 (11.5) | 3 (3.4) | 0 (0.0) | 21 (24.1) |  |
| **Health Insurance – Private, n(%)** | | | | | | | | 0.288 |
| Y | 555 (21.5) | 330 (59.5) | 64 (11.5) | 83 (15.0) | 25 (4.5) | 35 (6.3) | 18 (3.2) |  |
| N | 1906 (73.9) | 1079 (56.6) | 270 (14.2) | 266 (14.0) | 116 (6.1) | 119 (6.2) | 56 (2.9) |  |
| No response | 118 (4.6) | 69 (58.5) | 8 (6.8) | 15 (12.7) | 3 (2.5) | 1 (0.8) | 22 (18.6) |  |
| **Health Insurance – Medicare, n(%)** | | | | | | | | **<0.001** |
| Y | 1589 (61.6) | 975 (61.4) | 181 (11.4) | 207 (13.0) | 89 (5.6) | 85 (5.3) | 52 (3.3) |  |
| N | 919 (35.6) | 460 (50.1) | 157 (17.1) | 152 (16.5) | 54 (5.9) | 70 (7.6) | 26 (2.8) |  |
| No response | 71 (2.8) | 43 (60.6) | 4 (5.6) | 5 (7.0) | 1 (1.4) | 0 (0.0) | 18 (25.4) |  |
| **Health Insurance – Medicaid, n(%)** | | | | | | | | **<0.001** |
| Y | 333 (12.9) | 229 (68.8) | 31 (9.3) | 30 (9.0) | 9 (2.7) | 22 (6.6) | 12 (3.6) |  |
| N | 2138 (82.9) | 1184 (55.4) | 303 (14.2) | 323 (15.1) | 132 (6.2) | 132 (6.2) | 64 (3.0) |  |
| No response | 108 (4.2) | 65 (60.2) | 8 (7.4) | 11 (10.2) | 3 (2.8) | 1 (0.9) | 20 (18.5) |  |
| **Health Insurance – Tricare, n(%)** | | | | | | | | 0.076 |
| Y | 171 (6.6) | 107 (62.6) | 27 (15.8) | 15 (8.8) | 11 (6.4) | 6 (3.5) | 5 (2.9) |  |
| N | 2265 (87.8) | 1276 (56.3) | 306 (13.5) | 342 (15.1) | 127 (5.6) | 147 (6.5) | 67 (3.0) |  |
| No response | 143 (5.5) | 95 (66.4) | 9 (6.3) | 7 (4.9) | 6 (4.2) | 2 (1.4) | 24 (16.8) |  |
| **Health Insurance – VA, n(%)** | | | | | | | | 0.326 |
| Y | 223 (8.6) | 140 (62.8) | 26 (11.7) | 26 (11.7) | 13 (5.8) | 10 (4.5) | 8 (3.6) |  |
| N | 2251 (87.3) | 1270 (56.4) | 309 (13.7) | 331 (14.7) | 129 (5.7) | 145 (6.4) | 67 (3.0) |  |
| No response | 105 (4.1) | 68 (64.8) | 7 (6.7) | 7 (6.7) | 2 (1.9) | 0 (0.0) | 21 (20.0) |  |
| **Health Insurance – Indian Health Services, n(%)** | | | | | | | | **0.556** |
| Y | 9 (0.3) | 7 (77.8) | 1 (11.1) | 0 (0.0) | 1 (11.1) | 0 (0.0) | 0 (0.0) |  |
| N | 2427 (94.1) | 1374 (56.6) | 333 (13.7) | 354 (14.6) | 137 (5.6) | 155 (6.4) | 74 (3.0) |  |
| No response | 143 (5.5) | 97 (67.8) | 8 (5.6) | 10 (7.0) | 6 (4.2) | 0 (0.0) | 22 (15.4) |  |
| **Health Insurance – Other, n(%)** | | | | | | | | **0.419** |
| Y | 278 (10.8) | 169 (60.8) | 30 (10.8) | 36 (12.9) | 17 (6.1) | 16 (5.8) | 10 (3.6) |  |
| N | 2014 (78.1) | 1129 (56.1) | 286 (14.2) | 301 (14.9) | 115 (5.7) | 128 (6.4) | 55 (2.7) |  |
| No response | 287 (11.1) | 180 (62.7) | 26 (9.1) | 27 (9.4) | 12 (4.2) | 11 (3.8) | 31 (10.8) |  |

Table S2. Distribution of Internet savvy patients conditioning on portal usage frequency.

| Internet Savvy | Yes | No |  |
| --- | --- | --- | --- |
| Total | 1596 (61.9) | 983 (38.1) |  |
| **Portal usage level (times), n(%)** | | | *P* value |
| Level 1 (0) | 709 (44.4) | 769 (78.2) | < .001 |
| Level 2 (1 – 2) | 284 (17.8) | 58 (5.9) |  |
| Level 3 (3 – 5) | 312 (19.5) | 52 (5.3) |  |
| Level 4 (6 – 9) | 124 (7.8) | 20 (2.0) |  |
| Level 5 (10+) | 135 (8.5) | 20 (2.0) |  |
| No response | 32 (2.0) | 64 (6.5) |  |

Table S3. Distribution of outcomes depending on portal usage frequency.

| Outcomes | Total | Portal usage level (times) | | | | | *P* value |
| --- | --- | --- | --- | --- | --- | --- | --- |
|  |  | Level 1 (0) | Level 2 (1 – 2) | Level 3 (3 – 5) | Level 4 (6 – 9) | Level 5 (10+) |  |
| OwnAbilityTakeCareHealth | | | | | | | .02 |
| Not confident at all | 25 (1.0) | 16 (64.0) | 5 (20.0) | 2 (8.0) | 1 (4.0) | 1 (4.0) |  |
| A little confident | 110 (4.5) | 78 (70.9) | 7 (6.4) | 9 (8.2) | 7 (6.4) | 9 (8.2) |  |
| Somewhat confident | 620 (25.2) | 387 (62.4) | 69 (11.1) | 84 (13.5) | 43 (6.9) | 37 (6.0) |  |
| Very confident | 1151 (46.8) | 655 (56.9) | 180 (15.6) | 186 (16.2) | 67 (5.8) | 63 (5.5) |  |
| Completely confident | 553 (22.5) | 325 (58.8) | 79 (14.3) | 81 (14.6) | 24 (4.3) | 44 (8.0) |  |
| QualityOfCare | | | | | | | <.001 |
| Poor | 19 (0.8) | 9 (47.4) | 3 (15.8) | 1 (5.3) | 3 (15.8) | 3 (15.8) |  |
| Fair | 73 (3.2) | 48 (65.8) | 10 (13.7) | 10 (13.7) | 2 (2.7) | 3 (4.1) |  |
| Good | 393 (17.0) | 251 (63.9) | 59 (15.0) | 52 (13.2) | 15 (3.8) | 16 (4.1) |  |
| Very good | 925 (40.0) | 570 (61.6) | 109 (11.8) | 133 (14.4) | 61 (6.6) | 52 (5.6) |  |
| Excellent | 902 (39.0) | 466 (51.7) | 145 (16.1) | 153 (17.0) | 62 (6.9) | 76 (8.4) |  |
| ConfidenceGettingHealthInfo | | | | | | | .004 |
| Not confident at all | 33 (2.6) | 24 (72.7) | 5 (15.2) | 1 (3.0) | 1 (3.0) | 2 (6.1) |  |
| A little confident | 60 (4.7) | 46 (76.7) | 5 (8.3) | 4 (6.7) | 3 (5.0) | 2 (3.3) |  |
| Somewhat confident | 374 (29.0) | 247 (66.0) | 47 (12.6) | 46 (12.3) | 17 (4.5) | 17 (4.5) |  |
| Very confident | 503 (39.0) | 266 (52.9) | 84 (16.7) | 91 (18.1) | 32 (6.4) | 30 (6.0) |  |
| Completely confident | 319 (24.7) | 179 (56.1) | 42 (13.2) | 48 (15.0) | 23 (7.2) | 27 (8.5) |  |
| ConfidenceGettingCancerHealthInfo | | | | | | | .01 |
| Not confident at all | 20 (1.7) | 15 (75.0) | 2 (10.0) | 3 (15.0) | 0 (0.0) | 0 (0.0) |  |
| A little confident | 50 (4.3) | 41 (82.0) | 5 (10.0) | 2 (4.0) | 1 (2.0) | 1 (2.0) |  |
| Somewhat confident | 315 (27.3) | 190 (60.3) | 46 (14.6) | 48 (15.2) | 19 (6.0) | 12 (3.8) |  |
| Very confident | 408 (35.4) | 237 (58.1) | 49 (12.0) | 74 (18.1) | 20 (4.9) | 28 (6.9) |  |
| Completely confident | 359 (31.2) | 198 (55.2) | 56 (15.6) | 44 (12.3) | 27 (7.5) | 34 (9.5) |  |

**Detailed statistical procedure**

We derived a procedure that involves a set of conditional independence tests that could both address the confounding issue and determine the direction of the causal relationship. We tested two hypotheses for the whole population based on the graph model: one tests whether $T$ causes $Y$, also known as the expected causal direction, and the other tests whether $T$ is caused by $Y$, ie, the reverse causal direction.

In a typical setting, $Z$is binary, $Y$ is binary or ordinal, but $X_{1}$ can be a vector that contains all possible confounders in different formats (e.g., continuous and categorical). We have shown in [^1^] that, if and only if $T$ causes $Y$ exclusively for the whole population, then, $Z$ and $Y$ are not conditionally independent given $X_{1}$. However, when $X_{1}$ contains several confounders, the power of the conditional independence test is low. Therefore, instead of directly testing $Z\perp Y|X_{1}$, we propose a more efficient testing method. To accommodate several confounders, we proposed independence tests conditioning on the propensity score, a one-dimensional summary of the confounding effects, rather than all possible confounders to promote the power of identifying directional causal relationship. Due to this, we need to employ two statistical tests, i.e., Test A and B, for two complimentary hypotheses [^1^].

**Theorem A1**. Given $Z\perp Y|U$, and define $\pi_{U}:=P(Z=1|U)$, then, $Z \perp Y \left| U\to Z \perp Y \right|\pi_{U}$.

We introduce $\pi_{X_{1}}=P\left( Z=1 | X_{1} \right),$the probability that the IV takes value 1 given confounders $X_{1}$. This probability can be obtained using a logistic regression model with a lasso penalty. Then, according to Theorem A1, if we can show that given $\pi_{X_{1}}$, Z and Y are conditionally dependent, then, we can claim that given $X_{1}$, Z and Y are conditionally dependent. Since $\pi_{X_{1}}\in$ [0,1], we further discretize it into different levels based on its 20^th^, 40^th^, 60^th^, and 80^th^ percentile (denoted as $G_{1}$, $G_{2}$, $G_{3}$, $G_{4}$ and G_5_), and denoted this discrete variable as $\bar{\pi}_{X_{1}}$. Subsequently, the test of $Z\perp Y |\bar{\pi}_{X_{1}}$ (**denoted as Test B**) only concerns discrete variables and can be performed using the Conditional Mutual Information [^2^].

Further, Z and Y should be conditionally independent given both $T$ and $X_{1}$. We introduce the conditional probability $\pi_{X_{1},T}=P(Z=1|X_{1},T)$ and test $Z\perp Y|\bar{\pi}_{X_{1},T}$ (**denoted as Test A**). We have shown that, failing to reject Test A but rejecting Test B indicates existence of the expected causal relationship between the treatment and the outcome [^1^]. This statement is valid regardless of the existence of X_2_ (unobservable confounder). On the other hand, if $T$ is caused by $Y$ exclusively for the whole population, the opposite is true: failing to reject Test B but rejecting Test A, which indicates existence of the reverse causal relationship.

The robustness against different discretization using numerical experiments was investigated using numerical experiments. We chose the number of discretized groups using the following two metrics: cell size violation and power for each stratum. These two measures are based on Chi-squared tests, as the Conditional Mutual Information approach that we used for the conditional independence test is connected to Chi-squared independence test [^3^]. For the cell size rule in the Chi-square test, the expected value of the cell should be five or more in at least 80% of the cells, and the expected value of the cell being less than one is not allowed [^4^]. Increasing the number of strata allows for a finer categorization of patients, meaning that individuals within each group share more similar characteristics. However, this also increases the risk of violating cell size rules and decreases statistical power.

The results (shown in Table S5) suggest that using five discretization groups is a reasonable option. For outcomes with sufficient sample sizes, such as OATCH and QC, the pooled *P* values remained stable, and violations were infrequent. However, for the other two outcomes, CGHI and CGCHI, increasing the number of strata for the smaller sample sizes (n = 1289 and n = 1152, respectively) led to frequent violations and lower power. Overall, when the number of strata was five, violations were less frequent across all four outcomes, and the power did not deteriorate.

Table S5 Conditional independence tests by different numbers of strata.

| # of strata | OATCH | | | | | | QC | | | | | |
| --- | --- | --- | --- | --- | --- | --- | --- | --- | --- | --- | --- | --- |
|  | Test A | | | Test B | | | Test A | | | Test B | | |
|  | Pooled *P* value | cell size | power | Pooled *P* value | cell size | power | Pooled *P* value | cell size | power | Pooled *P* value | cell size | power |
| 4 | 0.024 | 1.000 | 0.457 | 0.035 | 1.000 | 0.450 | 0.038 | 1.000 | 0.466 | 0.002 | 1.000 | 0.609 |
| 5 | 0.056 | 1.000 | 0.393 | 0.024 | 1.000 | 0.477 | 0.046 | 0.918 | 0.434 | 0.004 | 1.000 | 0.536 |
| 6 | 0.051 | 1.000 | 0.407 | 0.041 | 1.000 | 0.440 | 0.051 | 0.857 | 0.421 | 0.002 | 1.000 | 0.487 |
| 7 | 0.072 | 1.000 | 0.376 | 0.033 | 1.000 | 0.446 | 0.087 | 1.000 | 0.371 | 0.006 | 0.996 | 0.488 |
| 8 | 0.040 | 1.000 | 0.396 | 0.035 | 1.000 | 0.431 | 0.048 | 0.997 | 0.376 | 0.007 | 0.951 | 0.414 |
| 9 | 0.117 | 0.999 | 0.344 | 0.052 | 0.999 | 0.414 | 0.063 | 0.982 | 0.367 | 0.014 | 0.767 | 0.432 |
| # of strata | CGHI | | | | | | CGCHI | | | | | |
|  | Test A | | | Test B | | | Test A | | | Test B | | |
|  | Pooled *P* value | cell size | power | Pooled *P* value | cell size | power | Pooled *P* value | cell size | power | Pooled *P* value | cell size | power |
| 4 | 0.074 | 1.000 | 0.450 | 0.058 | 1.000 | 0.419 | 0.237 | 1.000 | 0.344 | 0.043 | 1.000 | 0.459 |
| 5 | 0.102 | 0.996 | 0.405 | 0.072 | 1.000 | 0.375 | 0.344 | 1.000 | 0.310 | 0.105 | 1.000 | 0.382 |
| 6 | 0.269 | 0.970 | 0.309 | 0.013 | 1.000 | 0.456 | 0.346 | 1.000 | 0.307 | 0.139 | 0.907 | 0.373 |
| 7 | 0.073 | 0.990 | 0.380 | 0.016 | 0.997 | 0.406 | 0.386 | 0.933 | 0.288 | 0.103 | 0.896 | 0.366 |
| 8 | 0.035 | 0.865 | 0.405 | 0.046 | 0.982 | 0.362 | 0.288 | 0.970 | 0.307 | 0.092 | 0.751 | 0.367 |
| 9 | 0.034 | 0.748 | 0.385 | 0.056 | 0.929 | 0.350 | 0.370 | 0.871 | 0.290 | 0.140 | 0.663 | 0.349 |

**Alternative propensity score model**

We built an alternative model that included not only the original variable set but also all the possible interaction terms between any two variables in the penalized logistic regression model with a lasso penalty. This comprehensive variable inclusion improved the quality of the propensity score model and decreased the chance of model misspecification. In the comparison, we used the same conditional independence testing procedure and the same data. As the results in the main text (Table 1) using the simple propensity score model, we drew the same conclusions using the interaction propensity score model (see Table S6).

Table S6 Conditional independence tests for the unstratified cancer population using the propensity score model including the original variables and all the possible two-pair interactions.

|  | Test A^†^ | | Test B^‡^ | |
| --- | --- | --- | --- | --- |
| Outcomes^*^ | *Pooled P* value | Effect size | *Pooled P* value | Effect size |
| OATCH – OwnAbilityTakeCareHealth | .06 | .10 | .01 | .11 |
| QC – QualityOfCare | .03 | .10 | .01 | .13 |
| CGHI – ConfidenceGettingHealthInfo | .33 | .12 | .14 | .13 |
| CGCHI – ConfidenceGettingCancerHealthInfo | .44 | .10 | .08 | .15 |

^†Rejecting Test A is necessary for the reverse causal relationship.^

^‡Rejecting Test B is necessary for the expected causal relationship.^

^*Failing to reject Test A but rejecting Test B indicates existence of the expected causal relationship between the treatment and the outcome; the opposite indicates the reverse causal relationship. Rejecting both tests indicates existence of heterogenous causal relationships.^

**Reference**

1. Park J, Liang M, Alpert JM, Brown RF, Zhong X. The Causal Relationship Between Portal Usage and Self-Efficacious Health Information–Seeking Behaviors: Secondary Analysis of the Health Information National Trends Survey Data. *Journal of Medical Internet Research*. 2021;23(1):e17782. doi:10.2196/17782

2. Darbellay GA. An estimator of the mutual information based on a criterion for conditional independence. *Computational Statistics & Data Analysis*. 1999;32(1):1-17. doi:10.1016/S0167-9473(99)00020-1

3. Hoey J. The Two-Way Likelihood Ratio (G) Test and Comparison to Two-Way Chi Squared Test. Published online June 26, 2012. doi:10.48550/arXiv.1206.4881

4. McHugh ML. The Chi-square test of independence. *Biochem Med*. Published online 2013:143-149. doi:10.11613/BM.2013.018
